# Pediatric Cryptococcal Lymphadenitis in the Absence of AIDS: Case Report and Literature Review

**DOI:** 10.1155/2013/563081

**Published:** 2013-06-04

**Authors:** Fengchang Bao, Hongna Tan, Wei Liu, Yange Li, Huixia Li

**Affiliations:** ^1^Department of Hematology, Children's Hospital, Zhengzhou, 255 Gangdu Road, Henan 450052, China; ^2^Department of Radiology, The First Affilicated Hospital of Zhengzhou University, Zhengzhou, 1 Jianshe East Road, Henan 450000, China

## Abstract

We present a rare case of cryptococcal lymphadenitis without immunocompromization in a two-and-a-half-year-old child. He was referred to our center with a fifteen-day history of continued fever. Ultrasound and computed tomography (CT) revealed the enlargement of multiple lymph nodes and lung reticulonodular shadows. Hematological, immunological, and microbiological tests for hepatitis, lymphoma, AIDS, and immunoglobulin deficiencies were negative. Laboratory tests demonstrated elevated erythrocyte sedimentation rate, elevated plasma and urinary ß2-microglobulin (ß2-MG) levels, and elevated C-reactive protein and fibrinogen. Both blood routine and bone marrow aspiration showed elevated eosinophil granulocytes. The diagnosis of cryptococcal lymphadenitis was obtained by excisional biopsy of the cervical lymph nodes. The patient was treated with intravenous amphotericin B and oral flucytosine for five weeks, then with subsequent oral fluconazole for three months. The patient is now doing well. Our case suggests that the diagnosis of cryptococcal lymphadenitis is very difficult without etiology and pathology, especially for a patient with a normal immune system; lymph node biopsy is necessary to diagnose it, and immediate antifungal treatment is necessary to treat it.

## 1. Introduction

Cryptococcal lymphadenitis occurs commonly as an opportunistic infection in AIDS patients and may be life threatening [1–8]. Kim et al. reported one case of this infection in a patient with systemic lupus erythematosus [9]. This infection has several clinical but nonspecific manifestations, including pneumonia, meningitis, peritonitis, and disseminated infection. Cryptococcal lymphadenitis in the absence of AIDS is extremely rare, and it is difficult to distinguish from other lymphadenopathies such as lymphoma and tuberculosis. In the present case, we describe a pediatric case of cryptococcal lymphadenitis in a patient who did not have AIDS and who presented with multiple cervical and retroperitoneal lymph node enlargements and pulmonary inflammatory lesions. This case was misdiagnosed as lymphoma, and the final diagnosis was confirmed by cervical lymph node excisional biopsy. 

## 2. Case Presentation

A two-and-a-half-year-old child was referred to our center with a fifteen-day history of fever without an obvious remote cause. The child presented with a continued fever, with a maximum temperature of 40 degrees and without shivering, nausea, emesia, and hyperspasmia. The child was treated with two weeks of standard antibiotic treatment for pneumonia (indicated by a chest X-ray) in the local hospital before he came to our center; however, the fever was not controlled. At the time of his presentation, the child was fully conscious, and a history of contact with HIV was denied by his parents. Physical examination showed bilateral multiple cervical lymph node enlargements, as well as mild hepatosplenomegaly. A diagnosis of lymphoma was suspected by the clinician.

**Figure 1:**
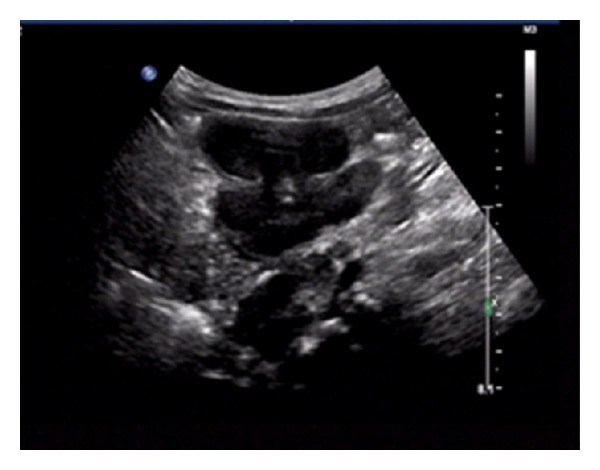
Ultrasound demonstrates multiple retroperitoneal lymph node enlargements.

**Figure 2:**
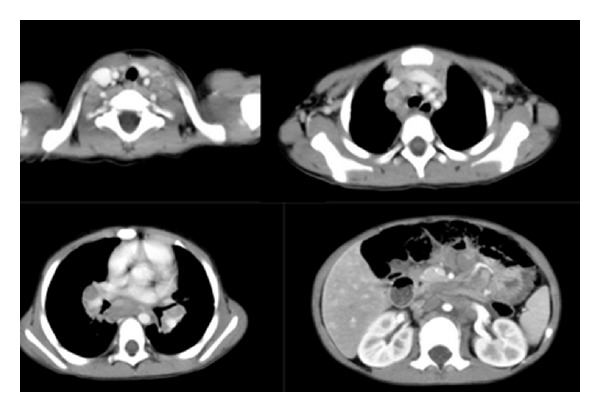
Thoracic and abdominal CT shows multiple lymph node enlargements in the regions of the bilateral neck and hilum, as well as the mediastinum and retroperitoneal areas.

**Figure 3:**
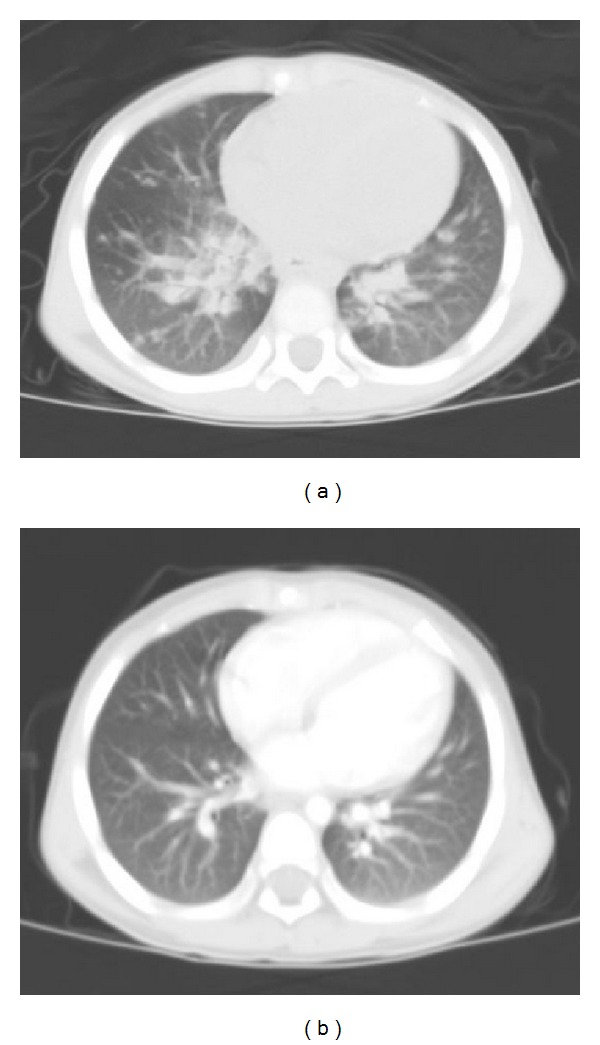
Thoracic CT reveals reticulonodular shadows in the lung window (a); after five weeks of treatment, thoracic CT showed that the lesions of the lung were reduced (b).

**Figure 4:**
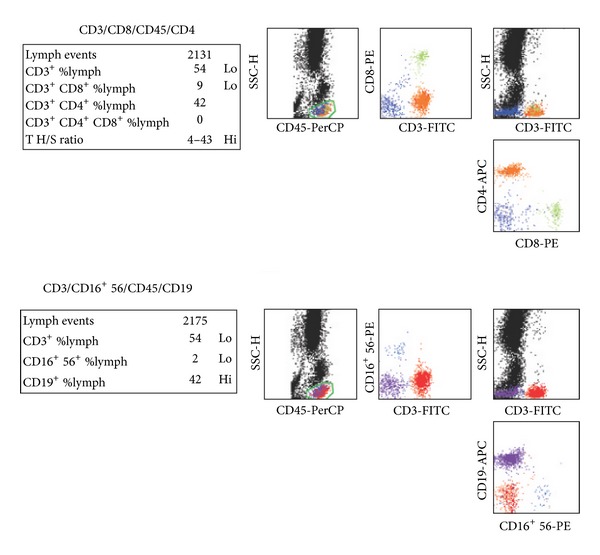
Lymphocyte subset detection by flow cytometry, which showed that the percentages of CD3, CD3/CD8^+^, CD3/CD4^+^, CD19^+^, and NK were 54%, 9%, 42%, 42%, and 2%, respectively.

**Figure 5:**
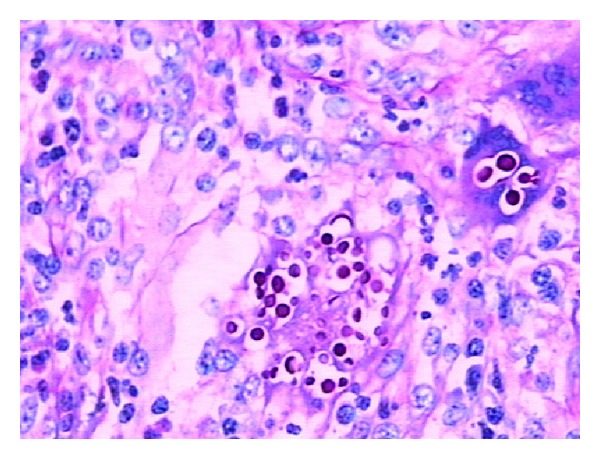
The cervical lymph node excisional biopsy revealed a lymphohistiocytic background with several blue, spherical structures that were surrounded by clear halos, which are indicative of Cryptococcus.

**Figure 6:**
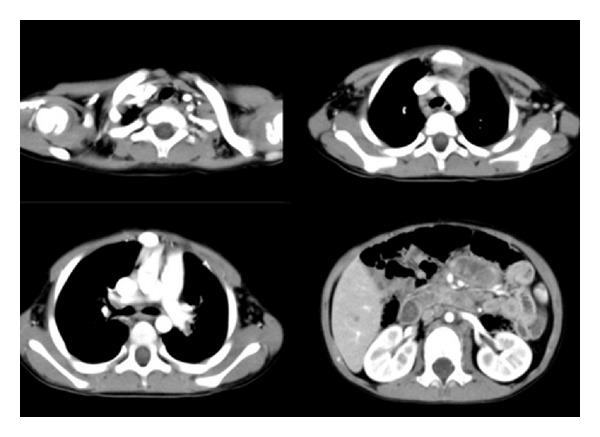
After five weeks of treatment, the thoracic and abdominal CT showed that the swelling of the lymph nodes was markedly diminished.

Detailed examinations were performed after he came to our center. Ultrasonography revealed multiple retroperitoneal lymph node enlargements (Figure 1). An enhanced 64-slice thoracic and abdominal computerized tomography (CT) scan was obtained, which revealed multiple lymph node enlargements in the bilateral neck, lung hilum, mediastinum, and retroperitoneal area (Figure 2), and some of the lymph nodes had fused into a mass and appeared as having a moderately homogeneous enhancement. At the same time, the thoracic CT scan also revealed reticulonodular shadows in the lung (Figure 3(a)). The ultrasound and CT findings could not exclude a diagnosis of lymphoma. *Cryptococcus neoformans* was not found by India ink stain of the cerebrospinal fluid. Hematological, immunological, and microbiological tests for hepatitis, lymphoma, AIDS, and immunoglobulin deficiencies were negative. Laboratory tests demonstrated an elevated erythrocyte sedimentation rate (20 mm/h), elevated plasma and urinary *β*2-MG levels (4.2 *μ*g/mL and 0.44 *μ*g/mL, resp.), elevated C-reactive protein (153.92 mg/L), and elevated fibrinogen (6.73 g/L). Blood routine showed elevated WBC (44.22 × 10^9^), neutrophil granulocytes (74.24%), eosinophil granulocytes (7.14%), and absolute counting of eosinophil granulocytes (3.13 × 10^9^). Bone marrow aspiration showed elevated eosinophil granulocytes (10.5%), and peripheral blood lymphocyte subset detection by flow cytometry showed that the percentages of CD3, CD3/CD8^+^, CD3/CD4^+^, CD19^+^, and NK were 54%, 9%, 42%, 42%, and 2%, respectively (Figure 4). Finally, a cervical lymph node excisional biopsy was performed, revealing a lymphohistiocytic background with several blue, spherical structures surrounded by clear halos, suggestive of Cryptococcus (Figure 5). The yeast-like structures were identified by periodic acid-Schiff (PAS) stain. After this patient was treated with intravenous amphotericin B (0.7 mg/kg/day) and oral flucytosine (100 mg/kg/day) for five weeks, the lung lesions and lymph nodes were markedly diminished in size (Figures 3(b) and 6). Subsequent oral fluconazole (12 mg/kg/day) was administered for three months, and the patient is now doing well.

## 3. Discussion

Cryptococcosis is a subacute to chronic infection caused by the encapsulated yeast *Cryptococcus neoformans*, which can involve the respiratory system, central nervous system, skin, lymph nodes, and other organs. Approximately 85% of patients with cryptococcosis have impaired cell-mediated immunity. AIDS-associated cryptococcal infections now account for 80–90% of all patients with cryptococcosis [10]. Cryptococcus neoformans is distributed worldwide; it can be inhaled into the respiratory tract, and depending on the immune response, the host may be asymptomatic or the disease may present as pneumonitis, pulmonary nodules, meningitis (if it disseminates to the CNS), or, less commonly, lymph node enlargement. Therefore, the respiratory tract and CNS are the primary sites of infection, with occasional involvement of the lymph nodes [11]. Cryptococcal lymphadenitis occurs more commonly in immunocompromised patients [5, 6, 8, 9]. Mitha et al. reported that HIV-negative patients generally present with pulmonary or CNS mass lesions, whereas meningoencephalitis is predominant in HIV-positive patients [11]. In our case, a pediatric HIV-negative patient with cryptococcal lymphadenitis presented with persistent high fever without obvious CNS symptoms, which is in agreement with previous studies. Although multiple lymph node enlargements are unusual in HIV-negative cryptococcosis, this phenomenon may be related to the patient's age. Our patient was two-and-a-half-year old, so it is possible that his immune system was not fully developed. To the best of our knowledge, this finding has not been previously reported.

The clinical appearances and laboratory tests for cryptococcal lymphadenitis are nonspecific. Indirect evidence of infection by detection of cryptococcal antigens is particularly helpful because the antigens have high sensitivity and specificity, but negative results do not absolutely rule out cryptococcosis because there may be only a small number of organisms present. In our patient, ultrasound and CT findings clearly demonstrated swollen lymph nodes regardless of neck or retroperitoneal, and thoracic CT also revealed the lung diseases. However, it was very difficult to distinguish cryptococcal lymphadenitis from lymphoma and tuberculosis in our case, despite the laboratory test results (elevated erythrocyte sedimentation rate, plasma and urinary *β*2-MG levels, and C-reactive protein and fibrinogen) [12–15]. Therefore, the diagnosis of cryptococcal lymphadenitis is generally based on lymph node biopsy [16, 17]. Histopathology will show cryptococcus as thin-walled, dark-staining cells with a visible artificial round halo, and some yeast forms have characteristic narrow-based buds; sometimes, special PAS stains can also be useful for detecting the organisms. Therefore, using the characteristic features revealed by pathology, cryptococcal lymphadenitis is diagnosed easily. Once cryptococcosis is positively diagnosed, immediate antifungal treatment is required. Intravenous amphotericin B and oral fluconazole are the most common therapies, but sometimes oral flucytosine is added [18].

Our case suggests that the diagnosis of cryptococcal lymphadenitis is very difficult without etiology and pathology. If both abnormal lymph nodes in the neck and inflammation in the lungs are confirmed in a child, cryptococcal lymphadenitis should be considered, even with a negative culture result. Lymph node biopsy should be performed, and if the diagnosis is established, antifungal treatment must be started as soon as possible to prevent complications related to cryptococcosis.
